# 1574. Co-Utilization of HIV, Substance Use, and Mental Health Services Among Women with Current Substance Use

**DOI:** 10.1093/ofid/ofad500.1409

**Published:** 2023-11-27

**Authors:** Ayako W Fujita, Aditi Ramakrishnan, Cyra Christina Mehta, Oyindamola Yusuf, Tracey Wilson, Steven Shoptaw, Adam Carrico, Adaora A Adimora, Ellen Eaton, Mardge H Cohen, Jennifer Cohen, Adebola adedimeji, Michael Plankey, Aruna Chandran, Deborah Jones Weiss, Jonathan Colasanti, Anandi N Sheth

**Affiliations:** EMORY UNIVERSITY SCHOOL OF MEDICINE, Atlanta, Georgia; Washington University in St. Louis, St. Louis, Missouri; Emory University School of Medicine, Atlanta, Georgia; Emory University School of Medicine, Atlanta, Georgia; Downstate Health Sciences University, Brooklyn, New York; University of California, Los Angeles, Los Angeles, California; University of Miami, Miami, Florida; Department of Medicine, University of North Carolina at Chapel Hill, Chapel Hill, North Carolina; University of Alabama, Birmingham, Birmingham, Alabama; Department of Medicine, Stroger Hospital of Cook County, Chicago, Illinois; University of California, San Francisco, San Francisco, California; Albert Einstein College of Medicine, Bronx, New York; Georgetown University, Washington, District of Columbia; Johns Hopkins University, Baltimore, Maryland; University of Miami Miller School of Medicine, Miami, Florida; Emory University School of Medicine, Atlanta, Georgia; Emory University School of Medicine, Atlanta, Georgia

## Abstract

**Background:**

Integrated HIV, mental health, and substance use (SU) treatment strategies to improve health outcomes among women living with HIV (WWH) are limited. We described co-utilization of HIV, SU, and mental health treatment services among women enrolled in the Women’s Interagency HIV Study (WIHS) who report current SU.

**Methods:**

We included data from participants enrolled in 10 WIHS sites during their last study visit from 2013-2020. Current SU was defined as self-reported, non-medical use of drugs in the past year, excluding use of only marijuana. We described utilization of each treatment service (SU treatment, HIV care, mental health care, alcohol use treatment, tobacco cessation treatment) by subgroups of participants with current SU and either HIV, depressive symptoms (Center for Epidemiologic Studies–Depression score ≥ 16), heavy alcohol use ( > 7 drinks/week), or current tobacco use. We then compared utilization of services by those who did or did not utilize SU treatment using Chi-square/Fisher exact tests.

**Results:**

Among women with current SU (n=377), 41.9% reported utilizing SU treatment (**Table**) and 82.7% receiving any health care. Among women with current SU+HIV (n=233), 86.3% had an HIV healthcare provider visit since last study visit; among current SU+depressive symptoms (n=204), 39.2% had a mental health provider visit since last visit; among current SU+heavy alcohol use (n=87), 23.0% utilized alcohol treatment in the past year; and among current SU+current tobacco use (n=296), 10.1% utilized tobacco cessation treatment in the past year. Among subgroups of women with current SU who were eligible for another service, utilization of other services was significantly higher among those who utilized SU treatment for alcohol and tobacco cessation treatment, but not for any healthcare provider, HIV care, or mental health care provider (**Figure**).

**Figure:**
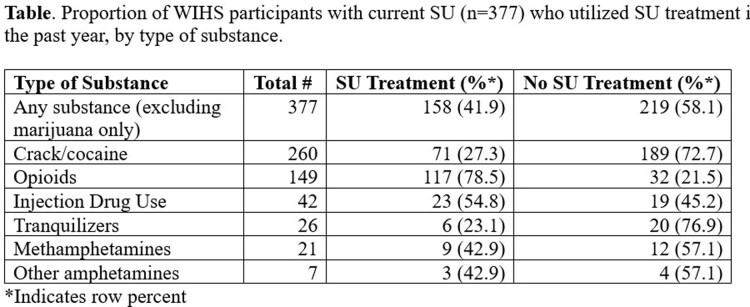

**Figure:**
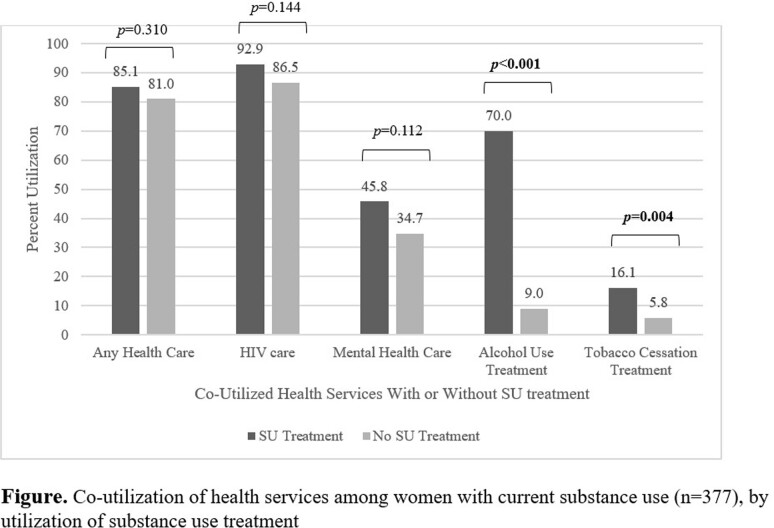

**Conclusion:**

Among this sample of WWH with current SU, we found 1) high engagement in SU treatment, and high engagement in health care and HIV care regardless of SU treatment, but 2) low engagement in alcohol and tobacco cessation treatments. Integrated drug, alcohol, and tobacco treatment programs should not be missed opportunities for WWH with concurrent SU, alcohol or tobacco use.

**Disclosures:**

**Cyra Christina Mehta, PhD, MSPH**, Merck: Grant/Research Support **Ellen Eaton, MD, MPH**, Gilead: Grant/Research Support|Gilead: Honoraria

